# Analysis of Serum Interleukin (IL)-1β and IL-18 in Systemic Lupus Erythematosus

**DOI:** 10.3389/fimmu.2018.01250

**Published:** 2018-06-07

**Authors:** Rachel Mende, Fabien B. Vincent, Rangi Kandane-Rathnayake, Rachel Koelmeyer, Emily Lin, Janet Chang, Alberta Y. Hoi, Eric F. Morand, James Harris, Tali Lang

**Affiliations:** Rheumatology Research Group, Centre for Inflammatory Diseases, School of Clinical Sciences at Monash Health, Monash University, Clayton, VIC, Australia

**Keywords:** biomarker, interleukin-1β, interleukin-18, lupus nephritis, organ damage, systemic lupus erythematosus

## Abstract

Systemic lupus erythematosus (SLE) is a chronic multisystem autoimmune disease characterized by biological and clinical heterogeneity. The interleukin (IL)-1 superfamily is a group of innate cytokines that contribute to pathogenesis in many autoimmune diseases. IL-1β and IL-18 are two members that have been shown to play a role in murine lupus-like models, but their role in human SLE remains poorly understood. Here, IL-1β and IL-18 were quantified by enzyme-linked immunosorbent assay in the serum of healthy controls (HCs) and SLE patients from a prospectively followed cohort. Disease activity and organ damage were assessed using SLE disease activity index 2000 (SLEDAI-2K) and SLE damage index scores (SDI), respectively. 184 SLE patients (mean age 44.9 years, 91% female, 56% double-stranded deoxyribonucleic acid positive) were compared to 52 HC. SLE patients had median [IQR] SLEDAI-2K of 4 [2,6], and SDI of 1 [0–2]. Serum IL-18 levels were statistically significantly higher in SLE patients compared to HCs. Univariable linear regression analyses showed that patients with active renal disease or irreversible organ damage had statistically significantly elevated serum IL-18 levels. The association between serum IL-18 and active renal disease was confirmed in multivariable analysis after adjusting for ethnicity and organ damage. High baseline serum IL-18 levels were associated with organ damage at the subsequent visit. Serum IL-1β levels were not significantly elevated in SLE patients when compared to HCs and had no association with overall or organ-specific disease activity or organ damage in cross-sectional and longitudinal analyses. Our data suggest that serum IL-18 and IL-1β have different clinical implications in SLE, with IL-18 being potentially associated with active renal disease.

## Introduction

Systemic lupus erythematosus (SLE) is a chronic, systemic autoimmune disease, characterized by biological and clinical heterogeneity (1). While survival rates have improved in the past 50 years, infection, cardiovascular disease, and lupus nephritis (LN) remain major causes of morbidity and mortality (2–4), and a recent study suggested no improvement in mortality over the last two decades (5). Dysregulation of both innate and adaptive immune responses have been implicated in the pathogenesis of SLE (6). Moreover, many cytokines have been shown to play a role in SLE, notably Type I Interferons (IFN) (7), B cell-activating factor (BAFF) (8), macrophage migration inhibitory factor (9, 10), and members of the interleukin (IL)-1 superfamily (9). Importantly, clinical studies associating patterns of expression with clinical disease have formed part of the evidence in support of successful clinical translation of treatments targeting BAFF and IFN (11).

Interleukin-1 family cytokines, including IL-1α, IL-β, and IL-18, are produced by innate immune cells such as macrophages and dendritic cells and share common aspects in their regulation, expression, and secretion (12). IL-1α and IL-1β are the best characterized members of the IL-1 family (12), while IL-18 has recently become of interest in relation to SLE (13). Studies using the MRL/lpr mouse model of lupus-like disease reported that increased IL-1β gene expression was associated with disease severity and accelerated disease progression (14, 15). Moreover, a study using the pristane-induced lupus-like model showed IL-1β deficient mice had significantly reduced levels of anti-double-stranded deoxyribonucleic acid (dsDNA) antibodies (Abs), serum pro-inflammatory cytokines, and disease activity when compared to both IL-1α deficient and control mice (16). In some human studies, a positive association between levels of serum IL-1β and disease activity has been observed (17–20), while others show no association (21–23). Only two clinical studies have investigated the associations of IL-1β with SLE clinical phenotypes; both reporting elevated levels of serum IL-1β associated with LN (20, 24). To date, no association between IL-1β with other clinical phenotypes in SLE has been reported.

Interleukin-18 has also been implicated in the pathogenesis of SLE in both mouse and human studies (25–27). MRL/lpr mice have increased levels of serum IL-18 compared to control animals and the administration of exogenous IL-18 to these mice worsened disease activity and nephritis (28). Moreover, IL-18-deficient mice or mice treated with anti-IL-18 in the MRL/lpr model show improved survival and decreased proteinuria when compared with controls (28–31). In human studies, levels of serum IL-18 have been reported to be increased in small studies of SLE patients compared to healthy controls (HCs) (32–34). Of interest, it has also been shown in some studies that levels of serum IL-18 positively correlate with SLE disease activity and is increased in patients with active disease (32, 34–36). Moreover, at the organ level, an association between levels of serum IL-18 and severity of LN has been identified (23, 34, 35, 37); however, this is not observed in all studies. No significant association with any other clinical phenotype, independent of renal activity, has been reported.

Despite being part of the same cytokine family, there are few clinical studies comparatively investigating IL-1β and IL-18 in SLE. Here, we examined the clinical associations of serum IL-1β and IL-18, particularly at the organ level, in a large, well-characterized prospectively followed SLE cohort. Using both univariable and multivariable regression analysis, we demonstrated a clear association between IL-18 and active renal disease.

## Materials and Methods

### Patients and Clinical Assessments

Adult patients attending the Monash Lupus Clinic (Melbourne, VIC, Australia) between June 2015 and July 2017 were recruited for this study. Patients recruited were also enrolled in the Australian Lupus Registry and Biobank (38). Patients were eligible if they fulfilled either the 1997 American College of Rheumatology (ACR) revised criteria (39) or the Systemic Lupus International Collaborating Clinic (SLICC) criteria (40). In this prospectively followed cohort, disease activity was assessed using the SLE disease activity index 2000 (SLEDAI-2K) at each clinical visit, as previously described (41, 42), and all routine clinical laboratory data and medication use were also recorded prospectively. Patients were classified as either having inactive (SLEDAI-2K ≤ 4) or active disease (SLEDAI-2K > 4), using the SLEDAI-2K cutoff integrated in the definition of a lupus low disease activity state (43). Organ-specific disease activity was determined by the presence of one or more positive scores in components of the SLEDAI-2K that pertain to different organ domains, as previously described (41). Renal SLEDAI-2K was defined using scores in the proteinuria, hematuria, pyuria, or urinary casts SLEDAI-2K descriptors (41, 44). Persistently active disease was defined as SLEDAI-K score > 4 at both baseline and follow-up visit. Organ damage was assessed at each annual visit using the SLICC-ACR Damage Index (SDI) score as described (43), and SDI > 0 was considered as connoting the presence of organ damage (45, 46). Organ-specific damage was assessed using the corresponding domain of the SDI score. Where historical or current renal biopsy data was available, histological classification of LN was determined using the World Health Organisation criteria (for biopsies performed before 2004) or the International Society of Nephrology and the Renal Pathology Society criteria (47). Median [inter-quartiles ranges; IQR] time interval between serum sample collection and renal biopsy was 2 [1, 7] years. Laboratory markers of SLE analyzed included hemoglobin (Hb), C-reactive protein (CRP), erythrocyte sedimentation rate (ESR), complement component 3 (C3), complement component 4 (C4), anti-dsDNA Ab, urine protein/creatinine ratio (UPCR), and estimated glomerular filtration rate (eGFR). All patients received standard-of-care therapy. Between February and April 2017, healthy adult individuals were enrolled as a HC group. Ethnicity in both cohorts was self-reported, as previously described in studies of ethnicity associations in SLE (48). Written, informed consent was obtained from all participants. Ethics approval for this project was obtained from Monash Health Human Research and Ethics Committee. The study was carried out in accordance with the National Statement of Ethical Conduct in Human Research (2007).

### Collection of Human Biological Samples

Whole blood samples were collected by venipuncture at routine clinical visits. Serum was isolated using serum-separating blood collection tubes and stored at −80°C, until further use.

### Serum Cytokine Quantification

Serum concentrations of IL-1β and total IL-18 were quantified by enzyme-linked immunosorbent assay (ELISA) (Human IL-1 ELISA MAX Deluxe, BioLegend, San Diego, CA, USA; Human total IL-18/IL-1F4 Quantikine ELISA kit, R&D Systems, Minneapolis, MN, USA), according to the manufacturers’ instructions. Samples with readings below the detection limit were assigned a value of 0.5 times the minimum detection value (IL-18: 5.85 pg/ml; IL-1β: 9.77 pg/ml). Given the low concentrations of IL-1β in serum, IL-1β levels were categorized as detectable or undetectable. Where indicated, analysis was also restricted to the subset of patients with detectable serum IL-1β when using serum IL-1β as a continuous variable.

### Statistical Analysis

Statistical analysis was performed using Stata 14.2 (StataCorp, College Station, TX, USA) software. Continuous variables were summarized as either mean (SD) or median [IQR] (range) depending on data distribution. Categorical data were summarized as number (frequency). For non-normally distributed variables, Wilcoxon rank-sum or Kruskal–Wallis tests were used to examine difference in two or more than two groups, respectively. Spearman’s rank correlation was used to assess correlations between two continuous variables. For normally distributed variables, *t* test or ANOVA were used to examine difference in two or more than two groups, respectively. Pearson’s chi-squared test or Fisher exact test were used to assess difference in proportions when appropriate. Serum IL-18 data were log10 transformed, and linear regression was used to examine associations of serum IL-18 with SLE clinical indicators for disease activity and organ damage. These linear regression analyses were repeated using the bootstrap method with 50 samples to derive confidence interval (CI) as a sensitivity analysis, since this method makes limited assumptions of the distribution of serum cytokine level. Variables with a *p*-value < 0.1 in the univariable linear regression analyses were included in the multivariable linear regression analysis. Potential collinearity between independent variables was assessed using a tetrachoric test before including them in the multivariable model. Results are presented as geometric means (GM) (antilogs of the means derived from linear regressions) and the ratios of GM (antilogs of regression coefficient) with corresponding 95% CI. Furthermore, serum IL-18 levels were categorized into a binary variable, using the median serum IL-18 levels as a cutoff, to allow for categorical analysis. Serum IL-18 concentrations lower or equal to the median were defined as low, while IL-18 concentrations greater than the median were defined as high. In addition, logistic regression was used to examine associations of serum IL-1β, categorized as a binary variable (detectable vs. non-detectable), with SLE clinical indicators for disease activity and organ damage. *p* values < 0.05 were considered statistically significant. *p* values lower than 0.01 were all defined as <0.01.

## Results

### Participant Characteristics

Data from 184 SLE patients were used in this study. Patient demographics and disease characteristics are outlined in Table 1. Briefly, the mean (SD) age and median [IQR] disease duration was 44.9 (14) and 10.2 [6, 17.2] years, respectively. Patients were predominantly female (91%), and approximately half were of Asian ethnicity. Approximately 38% of patients had active disease (SLEDAI-2K > 4) and approximately 58% had permanent organ damage (SDI > 0). Half of the patients were taking glucocorticoids at the time of the study, with a median [IQR] (range) dose among those taking glucocorticoids of 5 [5, 10] (1, 50) mg/day. A subset of 94 SLE patients were prospectively followed for a median [IQR] period of 1.1 [0.9, 1.4] years, and their disease characteristics summarized in Table S2 in Supplementary Material. Fifty-two HCs participated in this study, with a median [IQR] age of 36 [26.8, 44.1] years. Seventy-five percent (39/52) were female and 30% (16/52) were of Asian ethnicity. There was a statistically significant difference in age, gender and ethnicity between SLE patients and HC (Table S3 in Supplementary Material).

**Table 1:** SLE patient demographics and disease characteristics.

	SLE patients (*N* = 184)
**Demographics**	

Age (years), *mean (SD)*	44.9 (14)
Female, *n (%)*	167 (90.8%)
Asian ethnicity,^a^ *n (%)*	92 (51.4%)

**Clinical details**	

Disease duration (years), median [IQR] (range)	10.2 [6, 17.2] (0.6, 51.3)
SLEDAI-2K, median [IQR] (range)	4 [2, 6] (0, 28)
Patients with active disease (SLEDAI-2K > 4), *n* (%)	69 (37.5%)
Organ-specific disease activity	*N* (%)
Ocular	1 (0.5%)
Neuropsychiatric	1 (0.5%)
Renal	40 (21.7%)
Serositis	3 (1.6%)
Vasculitis	2 (1.1%)
Mucocutaneous	32 (17.4%)
Musculoskeletal	5 (2.7%)
Immunological	135 (73.8%)
Hematological	20 (10.9%)
Constitutional	2 (1.1%)
Biopsy-confirmed LN	58 (31.5%)
SLICC-SDI,^a^ median [IQR] (range)	1 [0, 2] (0, 7)
Patients with organ damage (SLICC-SDI > 0), *n* (%)	103 (57.5%)
Organ-specific damage	*N* (%)
Ocular	12 (6.7%)
Neuropsychiatric	29 (16.2%)
Renal	20 (11.2%)
Pulmonary	15 (8.4%)
Cardiovascular	21 (11.7%)
Peripheral vascular	15 (8.4%)
Gastrointestinal	4 (2.2%)
Musculoskeletal	39 (21.8%)
Skin	25 (14%)
Other^b^	20 (11.2%)

**Clinical laboratory data**	

Anti-dsDNA +ve, *n* (%)	103 (56%)
Complement (g/l)	Mean (SD)
C3	0.84 (0.26)
C4	0.16 (0.07)
Hemoglobin (g/l)	129.2 (15.5)
	Median [IQR] (range)
CRP (mg/l)	2 [0.7, 5] (0.2, 109)
ESR (mm/h)	13 [7, 26.5] (1, 125)
UPCR (g/mmol)^c^	0.02 [0.01, 0.04] (0, 9.14)
eGFR (ml/min/1.73m^2^)	90 [88.5, 90] (4, 93)

**Treatment**	***N* (%)**

Glucocorticoids	92 (50%)
Hydroxychloroquine	156 (84.8%)
Immunosuppressants^d^	106 (57.6%)
Biologics	3 (1.6%)

*Data are presented as medians [IQR] (range), mean (SD), or number (%) as indicated*.

*Anti-dsDNA, anti-double stranded DNA; C3, complement component 3; C4, complement component 4; CRP, C-reactive protein; eGFR, estimated glomerular filtration rate; ESR: erythrocyte sedimentation rate; IL, interleukin; LN, lupus nephritis; SLE, systemic lupus erythematosus; SLEDAI-2K, Systemic Lupus Erythematosus Disease Activity Index 2000; SLICC-SDI, Systemic Lupus International Collaborating Clinics—SLE Damage Index; UPCR, urine protein/creatinine ratio*.

*^a^Data missing for five patients*.

*^b^Includes premature gonadal failure, diabetes (regardless of treatment), and malignancy (excluding dysplasia) (46)*.

*^c^Data missing for one patient*.

*^d^Immunosuppressants include: methotrexate, azathioprine, leflunomide, cyclophosphamide and mycophenolate mofetil*.

### Serum IL-18 and IL-1β in SLE

**Figure 1:**
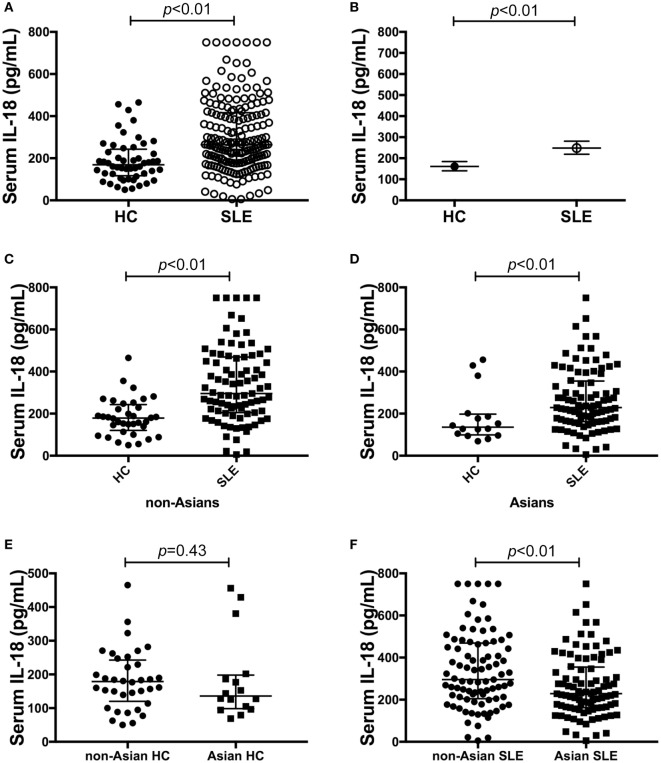
Association of serum interleukin (IL)-18 with systemic lupus erythematosus (SLE). **(A)** Serum IL-18 concentrations in healthy control (HC) (median [IQR]: 169 [117, 243] pg/ml; *n* = 52) vs. SLE patients (median [IQR]: 265 [178, 417] pg/ml; *n* = 184). **(B)** Geometric means (GM) of serum IL-18 in HC (GM (95%CI): 161 (140, 184) pg/ml; *n* = 52) vs. SLE patients [GM (95%CI): 248 (219, 281) pg/ml; *n* = 184] derived using univariable linear regression analysis. Ratio of the GMs was 1.54 with 95% CI between 1.3 and 1.84 with a *p*-value < 0.01. **(C)** Serum IL-18 concentrations in non-Asian HC (Median [IQR]: 179 [127, 238] pg/ml; *n* = 36) vs. non-Asian SLE patients (Median [IQR]: 296 [205, 469] pg/ml; *n* = 87). **(D)** Serum IL-18 concentrations in Asian HC (Median [IQR]: 136 [101, 194] pg/ml; *n* = 16) vs. Asian SLE patients (Median [IQR]: 229 [163, 352] pg/ml; *n* = 92). **(E)** Serum IL-18 concentrations in non-Asian HC (Median [IQR]: 179 [127, 238] pg/ml; *n* = 36) vs. Asian HC (Median [IQR]: 136 [101, 194] pg/ml; *n* = 16). **(F)** Serum IL-18 concentrations in non-Asian SLE (Median [IQR]: 296 [205, 469] pg/ml; *n* = 87) vs. Asian SLE (Median [IQR]: 229 [163, 352] pg/ml; *n* = 92). Panels **(A,C–F)** medians were compared using Mann–Whitney *U* tests.

Serum IL-18 was detectable in 99% (182/184) of SLE patients and all HC. SLE patients had statistically significantly higher median serum IL-18 than HC (median [IQR] of SLE vs. HC: 265 [178, 417] vs. 169 [117, 243] pg/ml; *p* < 0.01; Figure 1A). We also determined the GM of serum IL-18 in SLE patients and HC, and found that serum IL-18 in SLE patients was 54% greater than in HC (ratio of GM 1.54; 95% CI 1.3, 1.84; *p* < 0.01; Figure 1B). These results were not affected by reducing the HC to those matched by age, gender, and ethnicity (Tables S4 and S5 in Supplementary Material), and the association between increased serum IL-18 concentrations and SLE was further confirmed in multivariable analysis after adjusting for age, gender, and ethnicity (adjusted ratio of GM 1.56; 95% CI 1.19, 2.06; *p* < 0.01). The observed increase in serum IL-18 levels in SLE compared to HC was irrespective of the ethnic group (Figures 1C,D). Of note, serum IL-18 concentrations were statistically significantly higher in non-Asian compared to Asian SLE patients (Figures 1E,F; Table S6 in Supplementary Material). No statistically significant difference in serum IL-18 concentrations was seen between ethnic subsets of HC.

Serum IL-1β was undetectable in the majority of SLE and HC samples. The proportion of subjects with detectable serum IL-1β was statistically significantly higher in SLE patients [26.6% (49/184)] compared to HC [13.5% (7/52)] (*p* = 0.049). However, this result was not confirmed when analyzing the HC cohort limited to a subset matched with the SLE patient cohort characteristics (Table S4 in Supplementary Material). When the analysis was restricted to subjects with detectable IL-1β, no statistically significant difference in IL-1β levels were observed between SLE patients and HC (Figure S1A in Supplementary Material). We did not observe a statistically significant correlation between serum IL-18 and IL-1β levels in the subset of SLE patients with detectable serum IL-1β (*r* = −0.04; *p* = 0.81).

### Serum IL-18 and Organ-Specific Disease Activity and Damage

**Figure 2:**
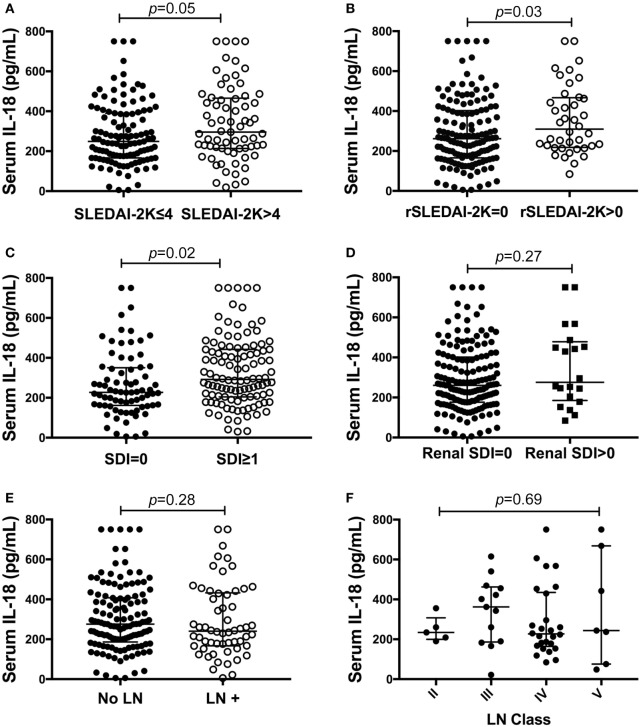
Association of serum interleukin (IL)-18 with renal systemic lupus erythematosus (SLE). **(A)** Serum IL-18 concentrations according to SLE disease activity [inactive disease (SLEDAI-2K ≤ 4, *n* = 116), vs. active disease (SLEDAI-2K > 4, *n* = 69)]. **(B)** Serum IL-18 concentrations in SLE patients according to renal disease activity [renal inactive (rSLEDAI-2K = 0, *n* = 144) vs. renal active (rSLEDAI-2K > 0: *n* = 40)]. **(C)** Serum IL-18 concentrations according to organ damage in SLE [organ damage absent (SLICC-SDI = 0); *n* = 76], vs. organ damage present (SLICC-SDI ≥ 1; *n* = 103). **(D)** Serum IL-18 concentrations according to renal organ damage in SLE [renal organ damage absent (renal SDI = 0); *n* = 159], vs. renal organ damage present (renal SDI ≥ 1; *n* = 20). **(E)** Serum IL-18 concentrations in SLE patients according to biopsy-confirmed lupus nephritis (LN) (no LN: *n* = 126 vs. LN: *n* = 58). **(F)** Serum IL-18 concentrations according to histological class of LN (II: *n* = 5, III: *n* = 13, IV: *n* = 26, V: *n* = 7). Serum IL-18 concentrations are expressed in picograms per milliliter. Medians were compared using Mann–Whitney *U* tests in panels **(A–E)**, and Kruskal–Wallis test in panel **(F)**. Horizontal bars indicate medians and corresponding error bars indicate inter-quartile ranges. SLICC-SDI, Systemic Lupus International Collaborating Clinic.

We next examined differences in serum IL-18 concentrations according to overall or organ-specific disease activity and damage. SLE patients with active disease had higher concentrations of IL-18 compared to patients with inactive disease (*p* = 0.05; Figure 2A). Patients with active renal disease had statistically significantly higher serum levels of IL-18 compared to those without renal activity (*p* = 0.03; Figure 2B). Moreover, serum IL-18 was weakly positively correlated with proteinuria (*r* = 0.16, *p* = 0.03) and weakly negatively correlated with eGFR (*r* = −0.18, *p* = 0.01). No significant differences in serum IL-18 concentrations according to other clinical phenotypic subsets were observed (Table S6 in Supplementary Material). Patients with organ damage had statistically significantly higher serum IL-18 than those without (Figure 2C). In light of elevated IL-18 with renal disease activity, we examined renal-specific damage and found no significant difference in serum IL-18 according to the presence or absence of renal damage (Figure 2D). Serum IL-18 levels showed a modest positive correlation with clinical laboratory markers of inflammation (ESR: *r* = 0.23, *p* < 0.01; CRP: *r* = 0.2, *p* < 0.01). A weak negative correlation was observed between serum IL-18 levels and Hb (*r* = −0.16, *p* = 0.035). No statistically significant correlation was found between serum IL-18 and levels of C3 and C4 (C3: *r* = −0.03; *p* = 0.72; C4: *r* = 0.03; *p* = 0.64). No statistically significant difference in serum IL-18 concentrations was seen according to the presence of anti-dsDNA Abs (Table S6 in Supplementary Material).

We further examined potential associations between serum IL-18 with demographics, disease activity, and organ damage using linear regression. Univariable regression analyses indicated that Asian SLE patients had statistically significantly lower serum IL-18 than non-Asian patients (*p* = 0.02). Patients with active renal disease had statistically significantly higher serum IL-18 when compared to patients without active renal disease [ratio of GM 1.37; 95% CI 1.14, 1.63; *p* < 0.01 (Table 2)]. Patients with proteinuria also had statistically significantly higher serum IL-18 concentrations when compared to those without proteinuria (ratio of GM 1.43; 95% CI 1.15, 1.78; *p* < 0.01) (Table 2). In addition, patients with organ damage had elevated GM serum IL-18 by 33% percent. No association between serum IL-18 and renal damage was observed (Table 2). Renal disease activity remained statistically significantly associated with increased serum IL-18 after adjusting for organ damage and ethnicity, both of which attenuated their association with serum IL-18 in the multivariable model (Table 3). Proteinuria was not included in the multivariable analysis due to strong collinearity with renal SLEDAI-2K (tetrachoric rho = 0.98). We further analyzed serum IL-18 in relation to biopsy-confirmed LN. No statistically significant difference in median or GM concentrations of serum IL-18 was shown according to the presence of biopsy-confirmed LN (Figure 2E; Table 2). There was also no difference in median serum IL-18 according to histological class of LN (Figure 2F). When the analysis was restricted to the biopsy-confirmed LN cohort, however, median and GM of serum IL-18 levels were confirmed to be statistically significantly increased in patients with active renal disease (Median [IQR]: 278 [213, 442] vs. 189 [124, 349] pg/ml; *p* = 0.03; ratio of GM 1.63; 95% CI 1.15, 2.31; *p* < 0.01). The correlation of serum IL-18 with proteinuria and eGFR were also stronger in the biopsy-confirmed LN cohort than in the whole cohort (*r* = 0.28, *p* = 0.03; and *r* = −0.44, *p* < 0.01, respectively).

**Table 2:** Univariable associations of serum IL-18 in systemic lupus erythematosus (SLE).

	Serum IL-18 (pg/ml) derived from univariable linear regression analyses				
	
Exposures	GM	(95% CI)	Ratio of GM	(95% CI)	*p*-Value
**Demographics**					

**Age**	–	–	1.01^a^	(1, 1.01)	0.2
**Gender**					
Females	247	(222, 275)	1.00		
Males	258	(205, 325)	1.04	(0.82, 1.33)	0.73
**Ethnicity**					
Non-Asians	276	(246, 310)	1.00		
Asians	218	(187, 255)	0.79	(0.65, 0.96)	0.02

**Clinical details**					

**Disease duration**	–	–	1.01^a^	(0.99, 1.02)	0.23
**SLEDAI-2K**					
SLEDAI-2K ≤ 4	233	(200, 272)	1.00		
SLEDAI-2K > 4	275	(237, 319)	1.18	(0.96, 1.45)	0.11
**Mucocut. SLEDAI-2K**					
Mucocut. SLEDAI-2K = 0	250	(225, 277)	1.00		
Mucocut. SLEDAI-2K > 0	240	(183, 314)	0.96	(0.72, 1.28)	0.78
**Immuno. SLEDAI-2K**					
Immuno. SLEDAI-2K = 0	238	(194, 293)	1.00		
Immuno. SLEDAI-2K > 0	251	(221, 285)	1.05	(0.85, 1.31)	0.64
**Haemato. SLEDAI-2K**					
Haemato. SLEDAI-2K = 0	249	(228, 272)	1.00		
Haemato. SLEDAI-2K > 0	235	(153, 361)	0.94	(0.6, 1.48)	0.80
**Renal SLEDAI-2K**					
Renal SLEDAI-2K = 0	232	(202, 266)	1.00		
Renal SLEDAI-2K > 0	316	(275, 364)	1.37	(1.14, 1.63)	<0.01
**Proteinuria**					
UPCR ≤ 0.05	229	(201, 261)	1.00		
UPCR > 0.05	328	(281, 384)	1.43	(1.15, 1.78)	<0.01
**LN**					
LN −ve	258	(231, 287)	1.00		
LN +ve	228	(181, 286)	0.88	(0.67, 1.16)	0.37
**SLICC-SDI**					
SLICC-SDI = 0	207	(165, 259)	1.00		
SLICC-SDI > 0	276	(245, 310)	1.33	(1.02, 1.74)	0.03
**Renal SDI**					
Renal SDI = 0	238	(212, 267)	1.00		
Renal SDI > 0	297	(246, 358)	1.25	(0.98, 1.58)	0.07

**Treatment**					

**Glucocorticoid**					
No	246	(206, 293)	1.00		
Yes	250	(214, 291)	1.02	(0.79, 1.3)	0.9
**HCQ**					
No	281	(225, 352)	1.00		
Yes	242	(213, 276)	0.86	(0.65, 1.13)	0.28
**Immunosuppressants**					
No	245	(211, 283)	1.00		
Yes	250	(217, 288)	1.02	(0.82, 1.27)	0.84

*^a^Regression coefficient*.

*Mucocut., immuno. and hemato. SLEDAI-2K stand for mucocutaneous, immunological and hematological SLEDAI-2K*.

*95% CI, 95% confidence interval; GM, geometric mean; HCQ, hydroxychloroquine; IL, interleukin; IS, immunosuppressants; LN, lupus nephritis; SLE, systemic lupus erythematosus; SLEDAI-2K, SLE Disease Activity Index 2000; SLICC-SDI, systemic Lupus International Collaborating Clinics—SLE Damage Index; UPCR, urine protein/creatinine ratio*.

**Table 3:** Multivariable associations of serum IL-18 in systemic lupus erythematosus (SLE).

	Serum IL-18 (pg/ml) derived from multivariable linear regression analyses				
	
Exposures	GM	(95% CI)	Ratio of GM	(95% CI)	*p*-Value
**SLICC-SDI**					
SLICC-SDI = 0	211	(168, 265)	1.00		
SLICC-SDI > 0	265	(233, 300)	1.25	(0.98, 1.6)	0.07
**Ethnicity**					
Non-Asians	266	(221, 320)	1.00		
Asians	218	(185, 258)	0.82	(0.65, 1.04)	0.10
**Renal SLEDAI-2K**					
Renal SLEDAI-2K = 0	224	(194, 260)	1.00		
Renal SLEDAI-2K > 0	308	(252, 377)	1.37	(1.09, 1.73)	<0.01

*95% CI: 95% confidence interval; GM: geometric mean; IL: interleukin; SLICC-SDI: systemic Lupus International Collaborating Clinics—SLE Damage Index; SLEDAI-2K, SLE disease activity index 2000*.

### Comparison of Low vs. High Serum IL-18 Subsets

We examined differences in patient demographics and disease characteristics in patients dichotomized according to serum IL-18 using the median value (Table S1 in Supplementary Material). In the subset of patients with high serum IL-18, the proportion of patients with Asian ethnicity was statistically significantly lower (*p* = 0.02), the proportion of patients with permanent organ damage was statistically significantly higher (*p* = 0.04), ESR was statistically significantly higher, while Hb was statistically significantly lower (*p* < 0.01 and *p* = 0.04, respectively). There was also a trend toward higher CRP levels in the subset of patients with high serum IL-18, although this was not statistically significant (*p* = 0.06) (Table S1 in Supplementary Material).

### Longitudinal Analysis of Serum IL-18 and SLE Clinical Features

We next examined the potential for baseline serum IL-18 to predict subsequent disease activity or organ damage using logistic regression. Univariable analysis revealed that high baseline serum IL-18 concentrations were associated with the presence of irreversible organ damage at the follow-up visit (OR 2.55; 95% CI 0.99, 6.55; *p* = 0.05). Baseline serum IL-18 concentrations were not associated with subsequent overall or renal disease activity (Table 4). Of note, the change from baseline serum IL-18 concentrations was weakly correlated with change in renal SLEDAI-2K (*r* = 0.21; *p* = 0.04), but not with any other clinical parameters measured (Table S9 in Supplementary Material).

**Table 4:** Longitudinal associations of baseline serum IL-18 and IL-1β concentrations with disease activity and organ damage.

	SLEDAI-2K > 4 subsequent visit			Persistently active disease			Renal SLEDAI-2K > 0 subsequent visit			Organ damage subsequent visit^a^		
				
Baseline serum cytokine	OR	(95% CI)	*P*-value	OR	(95% CI)	*P*-value	OR	(95% CI)	*P*-value	OR	(95% CI)	*P*-value
**Baseline IL-18**												
Baseline IL-18												
Low (≤median)	1.00			1.00			1.00			1.00		
High (>median)	1.87	(0.76, 4.59)	0.17	2.59	(0.93, 7.24)	0.07	1.09	(0.41, 2.88)	0.87	2.55	(0.99, 6.55)	0.05
Baseline IL-18												
First quartile (lowest)	1.00			1.00			1.00			1.00		
Second quartile	1.87	(0.55, 6.33)	0.32	0.95	(0.2, 4.43)	0.95	0.99	(0.28, 3.54)	0.99	1.47	(0.49, 4.4)	0.49
Third quartile	2	(0.59, 6.83)	0.27	1.88	(0.47, 7.45)	0.37	1.05	(0.29, 3.78)	0.94	2.53	(0.75, 8.48)	0.13
Fourth quartile (highest)	3.11	(0.86, 11.29)	0.08	3.6	(0.9, 14.39)	0.07	1.13	(0.28, 4.47)	0.87	3.65	(0.88, 15.11)	0.07
**Baseline IL-1β**												
Not detectable	1.00			1.00			1.00			1.00		
Detectable	0.86	(0.32, 2.26)	0.76	1.04	(0.35, 3.09)	0.94	1.39	(0.51, 3.8)	0.52	1.58	(0.6, 4.11)	0.35

*95% CI, 95% confidence interval; IL, interleukin; OR, odd ratio; SLE, systemic lupus erythematosus; SLEDAI-2K, SLE Disease Activity Index 2000; SLICC-SDI, systemic Lupus International Collaborating Clinics—SLE Damage Index*.

*^a^Defined as SLICC-SDI > 0*.

### Serum IL-1β and Organ-Specific Disease Activity and Damage

Serum IL-1β detectability was not associated with disease activity or organ damage in the cohort as a whole (Table S7 in Supplementary Material). Patients with detectable serum IL-1β had a statistically significant increase in median organ damage score compared to those without (*p* = 0.03) (Figure S1B and Table S8 in Supplementary Material). There was no significant difference in overall disease activity according to serum IL-1β (Figure S1C and Table S8 in Supplementary Material), and no significant correlation was found between serum IL-1β and SLEDAI-2K when restricting the analysis to patients with detectable IL-1β (*r* = 0.2; *p* = 0.17). SLE patients with detectable IL-1β had a statistically significantly higher ESR and lower Hb compared to those without (Table S8 in Supplementary Material). No statistically significant difference in other laboratory markers according to serum IL-1β detectability was observed (Table S8 in Supplementary Material).

### Longitudinal Analysis of Serum IL-1β and SLE Clinical Features

No statistically significant association was found between baseline serum IL-1β with subsequent disease activity or organ damage (Table 4). No statistically significant correlation was observed between change in serum IL-1β concentrations and change in SLE clinical parameters (Table S9 in Supplementary Material).

## Discussion

The IL-1 super family encompasses 11 members; some, including IL-1α, IL-1β, and IL-18, are known to be pro-inflammatory, while others are known more for their anti-inflammatory properties. IL-1α, IL-1β, and IL-18 are the most studied members of the IL-1 superfamily in the context of autoimmune disease and have all been variously reported to be involved in the pathogenesis of SLE (12, 13), although our own studies have also highlighted potential roles for IL-38 and, to a lesser extent, IL-37 (49, 50). In the present study, we show that SLE patients have significantly higher levels of serum IL-18, but not IL-1β, compared to HC. This finding has also been reported in a study by Amerio and colleagues (51). Our data also indicate a strong association between serum IL-18, though not IL-1β, with renal disease activity in SLE.

This work contributes a detailed analysis of clinical associations of IL-18 in a large and well-characterized prospectively followed cohort, using both univariable and multivariable analyses. Our results corroborate previous studies showing that IL-18 levels are significantly higher in SLE patients compared to HC (32–34). In particular, we report here an association of IL-18 with overall active disease, active renal disease, and organ damage, and that the association of IL-18 with active renal disease was retained after adjusting for other variables and also in the subset of patients with biopsy-proven LN. A previous study has similarly reported elevated levels of serum IL-18 in LN patients (33). In line with some previous studies, we observed no significant difference in serum IL-18 according to histological class of LN (23, 34). However, others have reported increased IL-18 in both the glomeruli and serum of LN class IV patients compared to classes III and V (33, 35, 37, 52). IL-18 has also been suggested as a potential predictive biomarker for long-term outcomes in pediatric LN (23). Renal disease is a major predictor of damage progression in SLE, and our findings of a near-significant association of IL-18 with organ damage in multivariable analysis (*p* = 0.07) are supported by previous findings in a smaller study which reported increased serum IL-18 in SLE patients with organ damage (53). Furthermore, we observed that high baseline serum IL-18 levels were associated with the presence of organ damage at the subsequent visit, suggesting a potential for serum IL-18 as a predictive biomarker for irreversible organ damage. Collectively, findings from these clinical studies, and improvement of LN in the setting of IL-18 deficiency or blockade in a lupus-prone mouse model, suggest a pivotal role for IL-18 in the pathogenesis of renal SLE. These findings potentially set the scene for the trialing of anti-IL-18 interventions in LN.

We did not observe elevated serum IL-1β in SLE compared to HC. Differences in findings from our study from some previous studies may reflect the fact that most studies in SLE have been performed in ethnically homogenous cohorts, as distinct from the ethnically diverse profile of the Monash cohort. Indeed, the role of IL-1β in SLE is contentious. In some studies, an association has been observed between serum IL-1β and disease activity, whilst other studies report no such association (19, 20, 22, 54). We found no significant association between serum IL-1β and overall or organ-specific SLE disease activity in cross-sectional and longitudinal analyses. However, we did find that patients with detectable serum IL-1β had higher organ damage scores. This is in contrast to the one previously published study investigating serum IL-1β and organ damage in SLE, which showed no difference in IL-1β levels according to SLICC-SDI, using the same cutoff (19). This difference may be explained by the low proportions of patients with organ damage in their cohort (19). Given the availability of potent IL-1 targeting biologic therapies approved in other human diseases (55), resolving the potential role of IL-1β in SLE is of potential importance. However, it may be the case that measurement of serum IL-1β is not the optimum approach to answer this question, as it may only be released transiently in the serum, or expressed locally in affected tissues or sites of inflammation. Unfortunately, gene expression signatures indicative of IL-1β release *in vivo* have not yet emerged in analysis of genome wide transcriptome studies to date (56).

Caveats to the interpretation of our study apply. First, although the study was prospectively conducted, it was performed in a single center. Second, the HC cohort was not age-, gender-, or ethnicity-matched to the SLE cohort. However, analysis of the HC cohort limited to a subset matched with the SLE patient cohort characteristics did not reveal any difference in the results regarding serum IL-18 analysis. Further, adjusting for these demographic variables using a multiple regression model did not reveal any difference in the results regarding serum IL-18 analysis. Some phenotypic subsets, such as active neurological or vasculitis, were too small to enable analysis of associations with serum cytokines. Finally, the number of patients with both active renal disease (as defined by SLEDAI-2K) and concurrent biopsy-confirmed LN was relatively small (*n* = 26), and the modest size of our biopsy-confirmed LN subset limited the analysis of renal histologic pattern. Future studies should enlist patients with active biopsy-confirmed LN in order to validate and further strengthen our findings.

In conclusion, our study suggests that serum IL-18 and IL-1β have different clinical associations in SLE, in particular highlighting the associations of IL-18 with active disease and damage, and the potential use of serum IL-18 as a biomarker or a therapeutic target in LN. At present, belimumab, an anti-BAFF therapy, is the sole approved biologic in SLE, showing modest efficacy (57, 58). However, in Phase III clinical trials leading to its approval, severe LN patients were excluded, highlighting the urgent unmet need to identify novel therapeutic targets for SLE in general and for LN in particular. Future investigations need to establish mechanisms by which IL-18 contributes to the pathogenesis of LN, and thereafter explore the potential for IL-18 to be targeted in SLE.

## Ethics Statement

Ethics approval for this project was obtained from Monash Health Human Research and Ethics Committee.

## Author Contributions

Each individual named as an author has made substantial contributions to the conception and design of the study, or acquisition of data, or analysis and interpretation of data. RM, EM, TL, and JH designed the experiments. FV, RK, JC, AH, and RK-R prepared patient clinical and healthy individual datasets. RM, EL, and TL performed experiments. RM, FV, and RK-R analyzed the data. RM, FV, EM, TL, and JH drafted the manuscript. All authors approved the final version of the manuscript to be submitted.

## Conflict of Interest Statement

The authors declare that the research was conducted in the absence of any commercial or financial relationships that could be construed as a potential conflict of interest.

## Abbreviations

Ab, antibody; ACR, American College of Rheumatology; ALRB, Australian lupus registry and biobank; BAFF, B cell-activating factor from the tumor necrosis factor family; C3, complement component 3; C4, complement component 4; CRP, C-reactive protein; dsDNA, double-stranded deoxyribonucleic acid; eGFR, estimated glomerular filtration rate; ELISA, enzyme-linked immunosorbent assay; ESR, erythrocyte sedimentation rate; GM, geometric mean; Hb, hemoglobin; HC, healthy control; IFN, interferon; IL, interleukin; IQR, inter-quartile range; ISN/RPS, International Society of Nephrology and the Renal Pathology Society; KO, knockout; LLDAS, lupus low disease activity state; LN, lupus nephritis; PAD, persistently active disease; SLE, systemic lupus erythematosus; SLEDAI-2k, SLE disease activity index 2000; SLICC, Systemic Lupus International Collaborating Clinic; SLICC-SDI, Systemic Lupus International Collaborating Clinics/ACR damage index; TNF, tumour necrosis factor; UPCR, urine protein/creatinine ratio; WHO, World Health Organisation.
