# Supplementary figures and images for: Analysis of Serum Interleukin (IL)-1β and IL-18 in Systemic Lupus Erythematosus
Source: Front Immunol. 2018 Jun 7;9:1250. doi: 10.3389/fimmu.2018.01250 (PMC5999794; doi:10.3389/fimmu.2018.01250)

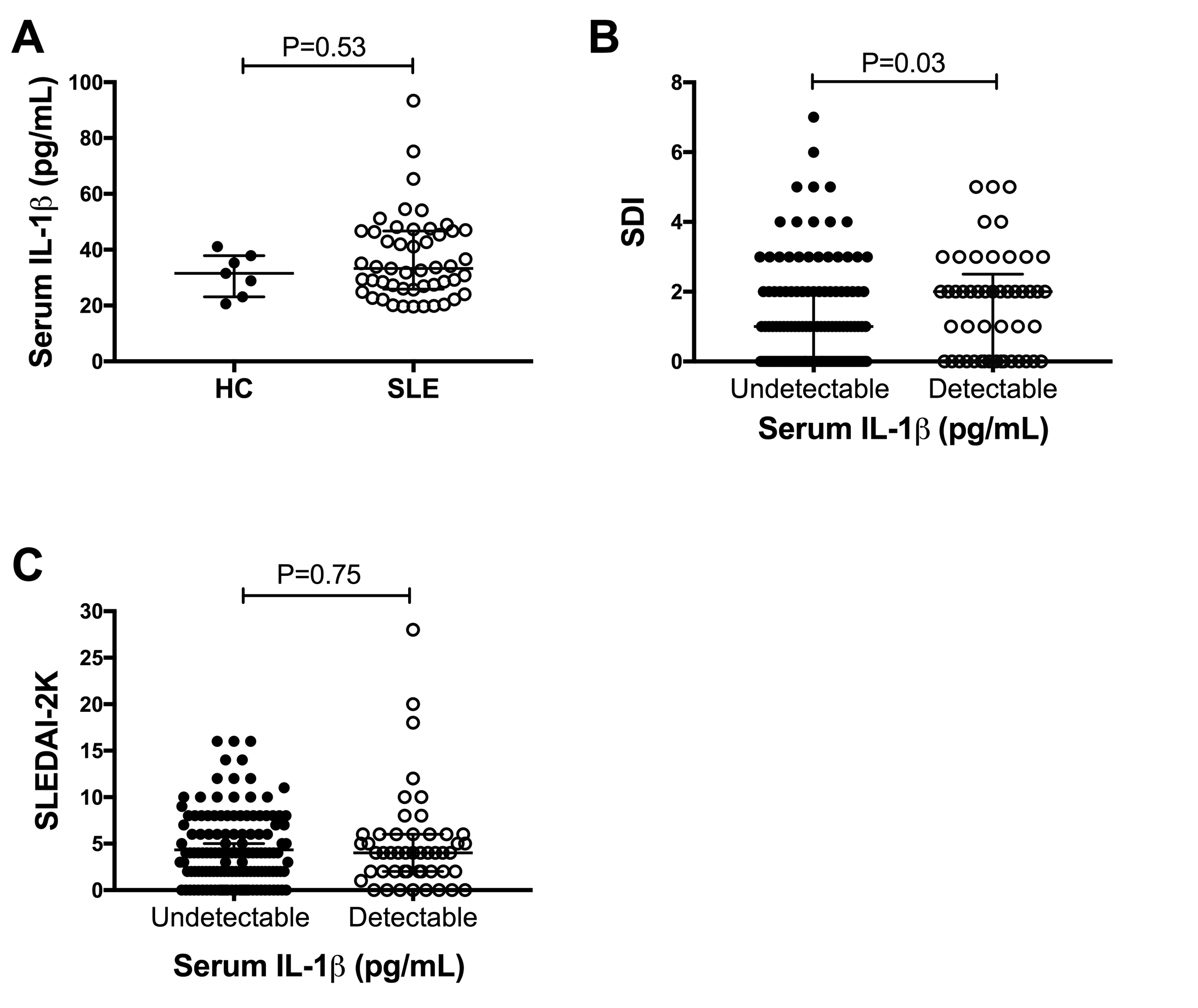

Supplement: Supplementary file 1 [file image_1.TIFF]
