# Supplementary material for: Analysis of Serum Interleukin (IL)-1β and IL-18 in Systemic Lupus Erythematosus
Source: Front Immunol. 2018 Jun 7;9:1250. doi: 10.3389/fimmu.2018.01250 (PMC5999794; doi:10.3389/fimmu.2018.01250)
Supplement: Supplementary file 2 [file table_1.DOCX]

**Analysis of Serum Interleukin (IL)-1β and IL-18 in Systemic Lupus Erythematosus**

**Mende *et al.***

**Supplementary Information**

**Supplementary Table 1. SLE patient demographics and disease characteristics according to median serum IL-18.**

|  | **Serum IL-18 ≤median** | **Serum IL-18 >median** | **P-value** |
| --- | --- | --- | --- |
|  | (n=92) | (n=92) |  |
| ***Demographics*** |  |  |  |
| **Age (years)***,* *mean (SD)* | 44.9 (13.7) | 44.9 (14.3) | 0.9 |
| **Female***, n (%)* | 83 (90%) | 84 (91%) | 0.8 |
| **Asians ethnicity***, n (%)* | 54 (60%) | 38 (43%) | 0.02 |
|  |  |  |  |
| ***Clinical details*** |  |  |  |
| **Disease duration (years)***, median [IQR]* | 9.2 [5, 17.2] | 11.2 [6.8, 17.2] | 0.1 |
| **SLEDAI-2K***, median [IQR]* | 4 (2, 6) | 4 (2, 6.5) | 0.37 |
| SLEDAI-2K >4*, n (%)* | 31 (34%) | 38 (41%) | 0.29 |
| **Renal SLEDAI-2K***, n (%)* | 18 (20%) | 22 (24%) | 0.47 |
| **Biopsy-confirmed LN** | 35 (38%) | 23 (25%) | 0.06 |
| **SLICC-SDI***, median [IQR]* | 0.5 (0, 2) | 1 (0, 3) | <0.01 |
| SLICC-SDI>0*, n (%)* | 46 (50%) | 57 (66%) | 0.04 |
| **Renal SDI***, n (%)* | 10 (11%) | 10 (11%) | 0.89 |
|  |  |  |  |
| ***Clinical Laboratory data*** |  |  |  |
| **Anti-dsDNA +ve***, n (%)* | 50 (54%) | 53 (58%) | 0.66 |
| **Complement (g/l)** | **Mean (SD)** | **Mean (SD)** |  |
| C3 | 0.84 (0.26) | 0.83 (0.27) | 0.7 |
| C4 | 0.16 (0.08) | 0.16 (0.07) | 0.99 |
| **Haemoglobin (g/l)** | 131.5 (12.8) | 126.8 (17.7) | 0.04 |
|  | **Median [IQR]** | **Median [IQR]** |  |
| **CRP (mg/l)** | 1.5 [0.7, 3.4] | 2.5 [0.7, 7] | 0.06 |
| **ESR (mm/h)** | 10 [6, 21.5] | 16 [9, 30.5] | <0.01 |
| **UPCR (g/mmol)** | 0.02 [0.01, 0.04] | 0.02 [0.01, 0.05] | 0.21 |
| **eGFR (ml/min/1.73m2)** | 90 [90, 90] | 90 [83, 90] | 0.21 |
|  |  |  |  |
| ***Treatment*** | **N (%)** | **N (%)** |  |
| **Steroids** | 46 (50%) | 46 (50%) | 1 |
| **Hydroxychloroquine** | 79 (86%) | 77 (84%) | 0.68 |
| **Immunosuppressants^***^** | 51 (55%) | 54 (59%) | 0.66 |

Data are presented as medians [IQR] (range), mean (SD) or number (%) as indicated.

Anti-dsDNA: Anti-double stranded DNA; C3: Complement component 3; C4: Complement component 4; CRP: C-reactive protein; eGFR, estimated glomerular filtration rate; ESR: Erythrocyte sedimentation rate; IL: Interleukin; LN: Lupus nephritis; SLE: systemic lupus erythematosus; SLEDAI-2K: Systemic Lupus Erythematosus Disease Activity Index 2000; SLICC-SDI: Systemic Lupus International Collaborating Clinics - SLE Damage Index; UPCR: urine protein/creatinine ratio.

^***^Immunosuppressants include: methotrexate, azathioprine, leflunomide, cyclophosphamide and mycophenolate mofetil.

**Supplementary Table 2. Disease characteristics of the SLE patient subset longitudinally followed.**

|  | **SLE subset longitudinally followed** |
| --- | --- |
|  | **(N=94)** |
| **Follow-up period (years)***, median [IQR] (range)* | 1.1 [0.9, 1.4] (0.4, 1.7) |
| **SLEDAI-2K***, median [IQR] (range)* | 4 [2, 6] (0, 16) |
| SLEDAI-2K >4*, n (%)* | 28 (30%) |
| Persistently active disease (PAD)*, n (%)* | 19 (20%) |
| **Renal SLEDAI-2K >0***, n (%)* | 22 (23%) |
| **SLICC-SDI***, median [IQR] (range)* | 1 [0, 2] (0, 6) |
| SLICC-SDI >0*, n (%)* | 61 (66%) |
| Change in SDI >0*, n (%)* | 10 (11%) |

Data are presented as medians [IQR] (range) or number (%) as indicated.

PAD: Persistently active disease; SLE: systemic lupus erythematosus; SLEDAI-2K: Systemic Lupus Erythematosus Disease Activity Index 2000; SLICC-SDI: Systemic Lupus International Collaborating Clinics - SLE Damage Index.

**Supplementary Table 3. Participant demographics in HC and SLE cohorts.**

|  | **HC** | **SLE** | **P-value** |
| --- | --- | --- | --- |
|  | **(N=52)** | **(N=184)** |  |
| **Age***, mean (SD)* | 36.2 (11) | 44.9 (14) | <0.01 |
| **Female***, n (%)* | 39 (75%) | 167 (90.8%) | <0.01 |
| **Asian ethnicity***, n (%)* | 16 (30.8%) | 92 (51.4%) | <0.01 |

HC: Healthy control; SLE: Systemic lupus erythematosus.

**Supplementary Table 4. Demographics and serum IL-18 and IL-1β in SLE patients and a matched subset of the HC cohort.**

|  | **Matched subset of HC** | **SLE** | **P-value** |
| --- | --- | --- | --- |
|  | **(N=36)** | **(N=184)** |  |
| ***Demographics*** |  |  |  |
| **Age***, mean (SD)* | 40.1 (10.8) | 44.9 (14) | 0.06 |
| **Female** | 30 (83.3%) | 167 (90.8%) | 0.18 |
| **Asians** | 14 (38.9%) | 92 (51.4%) | 0.17 |
| ***Serum cytokines*** |  |  |  |
| **IL-18***, median [IQR]* | 157 [98, 195] | 265 [178, 416] | <0.01 |
| **IL-1β detectability** | 6 (16.7%) | 49 (26.6%) | 0.21 |

Data are presented as medians [IQR], mean (SD) or number (%) as indicated.

HC: Healthy control; IL: Interleukin; SLE: Systemic lupus erythematosus.

**Supplementary Table 5. Univariable association of serum IL-18 and SLE using a matched subset of HC.**

|  | **Serum IL-18 levels (pg/mL)** | | | | |
| --- | --- | --- | --- | --- | --- |
|  | **derived from univariable linear regression analyses** | | | | |
| ***Exposures*** | **GM** | **(95% CI)** | **Ratio of GM** | **(95% CI)** | **P-value** |
| **Disease** |  |  |  |  |  |
| Matched subset of HC | 149 | (126, 177) | 1.00 |  |  |
| SLE | 248 | (221, 278) | 1.66 | (1.35, 2.04) | <0.01 |

95% CI: 95% Confidence Interval; GM: Geometric mean; IL: Interleukin; SLE: Systemic lupus erythematosus.

**Supplementary Table 6. Serum IL-18 concentrations according to demographics and clinical SLE parameters.**

|  |  | **SLE patients (N = 184)** | |
| --- | --- | --- | --- |
|  |  | **Serum IL-18 (pg/mL)** | |
|  | **n** | **Median [IQR]** | **P-value** |
| ***Demographics*** |  |  |  |
| **Age** |  |  | 0.49 |
| <40 | 81 | 269 [178, 423] |  |
| ≥40 & <60 | 78 | 251 [176, 395] |  |
| ≥60 | 25 | 277 [205, 442] |  |
| **Gender** |  |  | 0.84 |
| Female | 167 | 265 [176, 423] |  |
| Male | 17 | 253 [204, 355] |  |
| **Ethnicity** |  |  |  |
| Non-Asian | 87 | 296 [205, 469] | **<0.01** |
| Asian | 92 | 229 [163, 352] |  |
| ***Clinical Details*** |  |  |  |
| **SLEDAI-2k** |  |  | **0.05** |
| SLEDAI-2K≤4 | 115 | 249 [167, 390] |  |
| SLEDAI-2K>4 | 69 | 296 [214, 462] |  |
| **Renal SLEDAI-2K** |  |  | **0.03** |
| Renal SLEDAI-2K=0 | 144 | 262 [166, 401] |  |
| Renal SLEDAI-2K>0 | 40 | 310 [221, 466] |  |
| **Mucocut. SLEDAI-2K** |  |  | 0.82 |
| Mucocut. SLEDAI-2K=0 | 152 | 260 [180, 407] |  |
| Mucocut. SLEDAI-2K>0 | 32 | 285 [172, 462] |  |
| **Immuno. SLEDAI-2K** |  |  | 0.43 |
| Immuno. SLEDAI-2K=0 | 48 | 244 [178, 382] |  |
| Immuno. SLEDAI-2K>0 | 135 | 267 [178, 435] |  |
| **Haemato. SLEDAI-2K** |  |  | 0.65 |
| Haemato. SLEDAI-2K=0 | 163 | 264 [178, 407] |  |
| Haemato. SLEDAI-2K>0 | 20 | 298 [189, 449] |  |
| **Biopsy-confirmed LN** |  |  | 0.28 |
| Absent | 126 | 277 [199, 408] |  |
| Present | 58 | 240 [168, 429] |  |
| **SLICC-SDI** |  |  | **0.02** |
| SLICC-SDI=0 | 76 | 227 [162, 349] |  |
| SLICC-SDI>0 | 103 | 286 [200, 442] |  |
| **Renal SDI** |  |  | 0.27 |
| Renal SDI=0 | 159 | 261 [176, 402] |  |
| Renal SDI>0 | 20 | 276 [191, 470] |  |
| ***Laboratory Markers*** |  |  |  |
| **Proteinuria** |  |  | **<0.01** |
| No | 146 | 255 [166, 395] |  |
| Yes | 37 | 324 [234, 462] |  |
| **Anti-dsDNA +ve** |  |  | 0.39 |
| No | 81 | 253 [173, 404] |  |
| Yes | 103 | 266 [187, 424] |  |
| ***Treatments*** |  |  |  |
| **Glucocorticoids** |  |  | 0.78 |
| No | 92 | 266 [183, 407] |  |
| Yes | 92 | 264 [174, 420] |  |
| **Hydroxychloroquine** |  |  | 0.49 |
| No | 28 | 287 [171, 480] |  |
| Yes | 156 | 264 [182, 403] |  |
| **Immunosuppressants** |  |  | 0.66 |
| No | 78 | 264 [187, 369] |  |
| Yes | 106 | 270 [168, 442] |  |

Data are presented as medians [IQR].

Mucocut., immuno. and haemato. SLEDAI-2K stand for mucocutaneous, immunological and haematological SLEDAI-2K.

95% CI: 95% Confidence interval; Anti-dsDNA: Anti-double stranded DNA; GM: Geometric mean; IL: Interleukin; LN: Lupus nephritis; SLE: Systemic lupus erythematosus; SLEDAI-2K: SLE Disease Activity Index 2000; SLICC-SDI: Systemic Lupus International Collaborating Clinics - SLE Damage Index.

**Supplementary Table 7. Univariable associations of serum IL-1β in SLE.**

|  | **Detectable serum IL-1β** | | |
| --- | --- | --- | --- |
| ***Exposures*** | **OR** | **(95% CI)** | **P-value** |
| ***Demographics*** |  |  |  |
| **Age** | 0.99 | (0.97, 1.02) | 0.64 |
| **Gender** |  |  |  |
| Female | 1.00 |  |  |
| Male | 0.83 | (0.26, 2.69) | 0.76 |
| **Ethnicity** |  |  |  |
| Non-Asian | 1.00 |  |  |
| Asian | 0.93 | (0.48, 1.8) | 0.82 |
|  |  |  |  |
| ***Clinical details*** |  |  |  |
| **Disease duration** | 1.03 | (0.99, 1.06) | 0.15 |
| **SLEDAI-2k** |  |  |  |
| SLEDAI-2K≤4 | 1.00 |  |  |
| SLEDAI-2K>4 | 1.08 | (0.55, 2.11) | 0.83 |
| **Mucocut. SLEDAI-2K** |  |  |  |
| Mucocut. SLEDAI-2K=0 | 1.00 |  |  |
| Mucocut. SLEDAI-2K>0 | 1.86 | (0.83, 4.17) | 0.13 |
| **Immuno. SLEDAI-2K** |  |  |  |
| Immuno. SLEDAI-2K=0 | 1.00 |  |  |
| Immuno. SLEDAI-2K>0 | 1.54 | (0.7, 3.4) | 0.28 |
| **Haemato. SLEDAI-2K** |  |  |  |
| Haemato. SLEDAI-2K=0 | 1.00 |  |  |
| Haemato. SLEDAI-2K>0 | 0.9 | (0.31, 2.63) | 0.85 |
| **Renal SLEDAI-2K** |  |  |  |
| Renal SLEDAI-2K=0 | 1.00 |  |  |
| Renal SLEDAI-2K>0 | 0.63 | (0.27, 1.48) | 0.29 |
| **Proteinuria** |  |  |  |
| UPCR≤0.05 | 1.00 |  |  |
| UPCR>0.05 | 0.46 | (0.18, 1.19) | 0.11 |
| **LN** |  |  |  |
| LN -ve | 1.00 |  |  |
| LN +ve | 0.83 | (0.4, 1.69) | 0.6 |
| **SLICC-SDI** |  |  |  |
| SLICC-SDI=0 | 1.00 |  |  |
| SLICC-SDI>0 | 1.77 | (0.89, 3.52) | 0.11 |
| **Renal SDI** |  |  |  |
| Renal SDI=0 | 1.00 |  |  |
| Renal SDI>0 | 0.43 | (0.12, 1.55) | 0.2 |
|  |  |  |  |
| ***Treatment*** |  |  |  |
| **Glucocorticoid** |  |  |  |
| No | 1.00 |  |  |
| Yes | 1.32 | (0.69,2.55) | 0.41 |
| **Hydroxychloroquine** |  |  |  |
| No | 1.00 |  |  |
| Yes | 0.6 | (0.26, 1.41) | 0.24 |
| **Immunosuppressants** |  |  |  |
| No | 1.00 |  |  |
| Yes | 1.26 | (0.65, 2.46) | 0.49 |

Mucocut., immuno. and haemato. SLEDAI-2K stand for mucocutaneous, immunological and haematological SLEDAI-2K.

95% CI: 95% Confidence interval; IL: Interleukin; LN: Lupus nephritis; OR: Odd ratio; SLE: Systemic lupus erythematosus; SLEDAI-2K: SLE Disease Activity Index 2000; SLICC-SDI: Systemic Lupus International Collaborating Clinics - SLE Damage Index.

**Supplementary Table 8. SLE patient demographics and disease characteristics according to serum IL-1β detectability.**

|  | **Non-detectable**  **serum IL-1β** | **Detectable**  **serum IL-1β** | **P-value** |
| --- | --- | --- | --- |
|  | (n=135) | (n=49) |  |
| ***Demographics*** |  |  |  |
| **Age (years)***,* *mean (SD)* | 45.1 (14.2) | 44.1 (13.4) | 0.64 |
| **Female***, n (%)* | 122 (90.4%) | 45 (91.8%) | 0.9 |
| **Asians ethnicity***, n (%)* | 68 (51.9%) | 24 (50%) | 0.82 |
|  |  |  |  |
| ***Clinical details*** |  |  |  |
| **Disease duration (years)***, median [IQR]* | 9.2 ]5.1, 16.2] | 11.4 [7.9, 18.2] | 0.07 |
| **SLEDAI-2K***, median [IQR]* | 4 [2, 7] | 4 [2, 6] | 0.75 |
| SLEDAI-2K >4*, n (%)* | 50 (37%) | 19 (38.8%) | 0.83 |
| **Renal SLEDAI-2K***, n (%)* | 32 (23.7%) | 8 (16.3%) | 0.28 |
| **Mucocut. SLEDAI-2K***, n (%)* | 20 (14.8%) | 12 (24.5%) | 0.13 |
| **Immuno. SLEDAI-2K***, n (%)* | 96 (71.6%) | 39 (79.6%) | 0.28 |
| **Haemato. SLEDAI-2K***, n (%)* | 15 (11.2%) | 5 (10.2%) | 0.9 |
| **Biopsy-confirmed LN***, n (%)* | 44 (32.6%) | 14 (28.6%) | 0.6 |
| **SLICC-SDI***, median [IQR]* | 1 [0, 2] | 2 [0, 2] | **0.03** |
| SLICC-SDI>0*, n (%)* | 70 (53.8%) | 33 (67.3%) | 0.1 |
| **Renal SDI***, n (%)* | 17 (13.1%) | 3 (6.1%) | 0.29 |
|  |  |  |  |
| ***Clinical Laboratory data*** |  |  |  |
| **Anti-dsDNA +ve***, n (%)* | 73 (54.1%) | 30 (61.2%) | 0.39 |
|  |  |  |  |
| **Complement (g/l)***, mean (SD)* |  |  |  |
| C3 | 0.85 (0.25) | 0.79 (0.29) | 0.17 |
| C4 | 0.17 (0.08) | 0.15 (0.07) | 0.11 |
| **Haemoglobin (g/l)***, mean (SD)* | 131.2 (15.8) | 123.5 (13.3) | **<0.01** |
|  |  |  |  |
| **CRP (mg/l)***, median [IQR]* | 1.9 [0.6, 5] | 2.3 [0.9, 7] | 0.25 |
| **ESR (mm/h)***, median [IQR]* | 10 [6, 23] | 22 [13, 35] | **<0.01** |
| **UPCR (g/mmol)***, median [IQR]* | 0.02 [0.01, 0.04] | 0.02 [0.01, 0.04] | 0.9 |
| **eGFR (ml/min/1.73m2)***, median [IQR]* | 90 [87, 90] | 90 [90, 90] | 0.58 |
|  |  |  |  |
| ***Treatment*** |  |  |  |
| **Steroids***, n (%)* | 65 (48.1%) | 27 (55.1%) | 0.4 |
| **Hydroxychloroquine***, n (%)* | 117 (86.7%) | 39 (79.6%) | 0.24 |
| **Immunosuppressants^***^***, n (%)* | 76 (56.3%) | 30 (61.2%) | 0.55 |

Data are presented as medians [IQR], mean (SD) or number (%) as indicated.

Mucocut., immuno. and haemato. SLEDAI-2K stand for mucocutaneous, immunological and haematological SLEDAI-2K.

Anti-dsDNA: Anti-double stranded DNA; C3: Complement component 3; C4: Complement component 4; CRP: C-reactive protein; eGFR, estimated glomerular filtration rate; ESR: Erythrocyte sedimentation rate; IL: Interleukin; LN: Lupus nephritis; SLE: systemic lupus erythematosus; SLEDAI-2K: Systemic Lupus Erythematosus Disease Activity Index 2000; SLICC-SDI: Systemic Lupus International Collaborating Clinics - SLE Damage Index; UPCR: urine protein/creatinine ratio.

^***^Immunosuppressants include: methotrexate, azathioprine, leflunomide, cyclophosphamide and mycophenolate mofetil.

**Supplementary Table 9. Correlations between change from baseline (delta,** ∆**) in serum cytokine levels and SLE clinical parameters.**

|  | SLE subset longitudinally followed (N=94) | | | | |
| --- | --- | --- | --- | --- | --- |
|  |  | **∆ IL-1β** | | **∆ IL-18** | |
| *Clinical parameters* | **n** | **r value** | ***p* value** | **r value** | ***p* value** |
| ∆ SLEDAI-2K | 94 | -0.14 | 0.19 | 0.11 | 0.29 |
| ∆ Renal SLEDAI | 94 | -0.2 | 0.06 | 0.21 | **0.04** |
| ∆ SLICC-SDI | 93 | 0.0 | 0.9 | -0.04 | 0.7 |
| ∆ Anti-dsDNA | 91 | -0.12 | 0.26 | -0.02 | 0.88 |
| ∆ C3 (g/l) | 94 | -0.01 | 0.9 | -0.12 | 0.24 |
| ∆ C4 (g/l) | 94 | 0.003 | 0.9 | -0.09 | 0.4 |
| ∆ Haemoglobin (g/l) | 94 | -0.04 | 0.72 | -0.12 | 0.24 |
| ∆ CRP (mg/l) | 92 | -0.08 | 0.47 | 0.14 | 0.19 |
| ∆ ESR (mm/h) | 93 | -0.15 | 0.15 | 0.15 | 0.16 |
| ∆ UPCR (g/mmol) | 94 | -0.09 | 0.39 | 0.14 | 0.17 |
| ∆ eGFR (ml/min/1.73m2) | 94 | 0.16 | 0.13 | -0.06 | 0.59 |

Data are presented as number (%) or Spearman’s correlation test r and *p* values where indicated.

Anti-dsDNA: Anti-double stranded DNA; C3: Complement component 3; C4: Complement component 4; CRP: C-reactive protein; eGFR, estimated glomerular filtration rate; ESR: Erythrocyte sedimentation rate; IL: Interleukin; SLEDAI-2K: Systemic Lupus Erythematosus Disease Activity Index 2000; SLICC-SDI: Systemic Lupus International Collaborating Clinics - SLE Damage Index; UPCR: urine protein/creatinine ratio

**Supplementary Figure 1. Serum IL-1β** **in SLE.**

**(A)** Serum levels of IL-1β in HC (Median [IQR]: 32 [23, 38] pg/ml; n=7) versus SLE (Median [IQR]: 33 [26, 47] pg/ml; n=49), when restricting the analysis to subsets of individuals with detectable serum IL-1β. **(B)** SLICC-SDI score in subset of patients with undetectable serum IL-1β (Median [IQR]: 1 [0, 2]; n=130) vs. subset of patient with detectable serum IL-1β: (Median [IQR]: 2 [0, 2.5]; n=49). **(C)** SLEDAI-2K score in subset of patients with undetectable serum IL-1β (Median [IQR]: 4 [2, 7]; n=135) vs. subset of patient with detectable serum IL-1β: (Median [IQR]: 4 [2, 6]; n=49).

Medians were compared using Mann Whitney U tests in panels A to C. Horizontal bars indicate medians and corresponding error bars indicate inter quartile ranges.
